# Susceptible and Protective HLA Class 1 Alleles against Dengue Fever and Dengue Hemorrhagic Fever Patients in a Malaysian Population

**DOI:** 10.1371/journal.pone.0013029

**Published:** 2010-09-28

**Authors:** Ramapraba Appanna, Sasheela Ponnampalavanar, Lucy Lum Chai See, Shamala Devi Sekaran

**Affiliations:** 1 Department of Medical Microbiology, Faculty of Medicine, University Malaya, Kuala Lumpur, Malaysia; 2 Department of Infectious Diseases, University Malaya Medical Centre, Kuala Lumpur, Malaysia; 3 Department of Pediatrics, Faculty of Medicine, University Malaya, Kuala Lumpur, Malaysia; Global Viral Forecasting Initiative, United States of America

## Abstract

**Background:**

The human leukocyte antigen alleles have been implicated as probable genetic markers in predicting the susceptibility and/or protection to severe manifestations of dengue virus (DENV) infection. In this present study, we aimed to investigate for the first time, the genotype variants of HLA Class 1(-A and -B) of DENV infected patients against healthy individuals in Malaysia.

**Methodology/Principal Findings:**

This study was carried out with 92 dengue disease patients and 95 healthy controls from three different ethnic groups (Malay, Chinese and Indian) in Malaysia. All patients with clinical and laboratory confirmation of DENV infection were typed for the HLA-A and B loci, using polymerase chain reaction-sequence specific primer techniques. In our total population, a significant increase for HLA-B*53 (*P* = 0.042, *Pc* = 1.008) allele and a significant decrease for A*03 (*P* = 0.015, *Pc* = 0.18, OR = 5.23, 95% CI = 1.19–23.02) and B*18 (*P* = 0.017, *Pc* = 0.408) alleles were noted in DHF patients as compared to healthy donors. We also observed that in the Malay DHF patients, allele B*13 (*P* = 0.049, *Pc* = 1.176, OR = 0.18, 95% CI = 0.03–0.90) was present at a significantly higher frequency in this population while allele HLA-B*18 (*P* = 0.024, *Pc* = 0.576) was seen to be negatively associated with DHF.

**Conclusions/Significance:**

These are the first findings on genetic polymorphisms in our population and we conclude that: (1) In our total population, HLA-B*53 probably involve in disease susceptibility, while the HLA-A*03 and HLA-B*18 may confer protection from progression to severe disease; (2) In the Malay population, HLA-B*13 and B*18 are probably associated in disease susceptibility and protection, respectively. These results could furnish as a valuable predictive tool to identify ethnically different individuals at risk and/or protection from severe forms of DENV infection and would provide valuable informations for the design of future dengue vaccine.

## Introduction

Malaysia consists of two geographical regions divided by the South China Sea (latitude 2°30′ N, longitude 112°30′ E), with a population of 27.5 million people, consisting of multi-ethnic groups (Malay 53.3%, Chinese 26.0%, Indigenous 11.8%, Indian 7.7%, others 1.2%) [1]. History has revealed that Malaysia's geographical position places it between the great civilizations of Europe and the Middle East to the West and China and Japan to the East. This has made Malaysia a natural meeting place of trade routes and ultimately formed a multi-racial and multi-cultural nation [2].

The existence of varied human populations in Malaysia implies diverse genetic diversity, such as in the human leukocyte antigen (HLA) alleles. HLA is encoded by the major histocompatibility complex (MHC) and is located on the chromosome 6 in humans [3]. Both class I and II molecules are involved in displaying peptide antigen to host T lymphocytes in order to activate immune response [4]. However, the interaction between antigenic epitopes and the host immune system varies with the HLA allele involved [5]. This information on HLA alleles would probably be useful in identifying appropriate epitopes for inclusion in molecular vaccines and in determining the possible efficacy of these vaccines in a particular population [6].

Dengue viruses (DENVs) belong to the genus flavivirus, family *Flaviviridae* and are subgrouped into four serotypes: (DENV-1 to DENV-4) [7], [8]. This virus causes dengue fever (DF), dengue hemorrhagic fever (DHF) and dengue shock syndrome (DSS) which are identified as the most extensive vector-borne viral disease in humans. The virus is prevalent in tropical and sub-tropical regions around the world, predominantly in urban and semi-urban areas. Dengue fever has been endemic in Malaysia since 1901 [9] and reached epidemic proportions in 1973 [10]–[13]. In 1982, Malaysia experienced a major dengue/dengue hemorrhagic fever outbreak, which has affected all states in Peninsular and East Malaysia [14]. Since than, dengue has became a major public health problem especially among the highly urbanized states in Malaysia.

Almost 50% of the infections are recognized to be clinically silent infections [15]. In about 5–30% of the cases, the disease can be severe and complicated, with the symptoms of thrombocytopenia, plasma leakage, bleeding, and hypovolemic shock commonly referred to as DHF and DSS [16], [17]. The phenomenon of Antibody Dependent Enhancement (ADE) theory postulates that the infection with one dengue serotype during primary infection confers future protective immunity against that particular serotype but not with other serotypes during a secondary infection [18].

In addition to the ADE hypothesis, viral virulence and interferon (IFN) mediated immunopathogenesis are insufficient to explain clinical manifestations which have been implicated in the pathogenesis of DHF. Others have suggested that, host genetic factors, such as HLA alleles, play an important role in susceptibility or protection in dengue viral infections [19], [20]. Limited work has been established on the role of classical HLA-A and -B alleles in determining resistance, susceptibility, or the severity of acute viral infections in diverse populations worldwide. Previous studies have suggested that polymorphisms especially in this class I region gene were found to be associated with DHF disease both in susceptibility and protection. However, these associations also might vary by ethnic and geographical distribution. Study on DENV infected Thai and Cuban patients suggested that the classical class I serotype HLA-A2 was found to be in higher frequency in Thai DHF patients but this association was not seen in the Cuban study [19], [20]. More recently, two different molecular analysis of HLA class I allele profiles in Vietnamese and Cuban patients have provided evidence for association of various HLA class 1 loci with susceptibility to DHF [21], [22].

To our knowledge, the frequencies of HLA class 1 alleles in Malaysian population infected with DENV have not been documented previously. Thus, this study aimed to analyze the frequency, variation and specific polymorphism in HLA class I, A and B regions in DENV infected patients in the three ethnic groups (Malay, Chinese and Indian) in Malaysia. This would be an effort to determine the mechanisms of certain alleles that could be a contributing factor in susceptibility and/or protection against DENV infection in our population.

## Materials and Methods

### Study population

The study was carried out from January 2005 to June 2008. Blood samples were obtained from 41 (14 Malay, 14 Chinese and 13 Indian) clinically diagnosed adult patients with DF and 51 (19 Malay, 16 Chinese and 16 Indian) adult patients with DHF who were admitted to the University Malaya Medical Centre (UMMC) Kuala Lumpur, Malaysia. Three ml of blood was collected from each patient each in EDTA containing tube and in a plain tube. Patients with DF and DHF were classified according to World Health Organization (WHO) criteria (WHO, 2009). Age, gender, race and medical history were recorded for each patient. Clinical information including ascites, pleural effusion and circulatory disturbance as a result of plasma leakage were collected to enable disease classification. Platelet counts and hematocrit values were recorded serially during hospitalization. As for the background population for this genetic study, five ml of blood was retrieved in an EDTA containing tube and also in a plain tube from 95 unrelated healthy donors (32 Malays, 32 Chinese and 31 Indians), with no history of DENV infection. Only patients and donors with both parents were from the same race were included in this study.

### Ethics Statement

Written informed consent was obtained from the patients and donors. Ethical clearance has been approved by the Scientific and Ethical Committee at the University Malaya Medical Centre (UMMC) (Ethics Committee/IRB Reference No: 321.4).

### Dengue virus PCR and serology

Dengue virus RNA was extracted from serum samples using the QIAamp^R^ Viral RNA mini kit (Qiagen). RNA was reverse transcribed and a one step real-time RT-PCR assay was performed using SYBR-Green technology for detection of four different DENV serotypes [23]. All samples were further confirmed for DENV infection serologically using an in-house capture IgM Enzyme-Linked Immunosorbent Assay (ELISA) [24]. The primary and secondary DENV infection were defined based on the IgG antibody titers determined by a haemagglutination inhibition (HI) test in paired acute and convalescent sera [25].

### Human DNA extraction

Human DNA was extracted from whole blood using the AccuPrep^R^ Genomic DNA extraction kit (Bioneer, USA) following the manufacturer's instructions. Briefly, 200µl of whole blood were added to each tube containing 20 µl Proteinase K. Following that, 200 µl of binding buffer was added into this mixture and resuspended to achieve maximum lysis efficiency and incubated at 60°C for 10 minutes. After the addition of 100µl isopropanol, the lysate was transferred to the upper reservoir of the binding column tube and centrifuged at 8000 rpm for 1 minute. The reservoir was then transferred to new tube and washed twice with washing buffer 1 and 2 and centrifuged at 8000 rpm for 1 minute. After final centrifugation at 12,000 rpm for 1 minute, DNA was eluted in 200 µl of elution buffer by centrifugation at 8000 rpm for 1 min in a new tube and frozen in −20°C till use.

### HLA typing

HLA types of all the study subjects were determined using a sequence-specific primers (SSP) for HLA-A (Lot No: R53, X92, 26F) Combi Tray (24 tubes) and HLA-B (Lot No: V30, X82, Y52, 76E) Combi Tray (48 tubes) (Olerup SSP™, Saltsjobaden, Sweden) according to the manufacturer's instructions. The HLA-A*24 subtyping was performed using the SSP for HLA-A*24 high resolution (Lot No: X96, 63E) Combi Tray (48 and 76 tubes) (Olerup SSP™, Saltsjobaden, Sweden). Olerup SSP HL-A, HLA-B and HLA-A*24, PCR master mix complete with nucleotides, buffer, glycerol and cresol red were used for the PCR reaction. The DFS-taq DNA polymerase (Bioron GmbH) was added separately to the reaction tube. PCR amplification was carried out in a 96 well thermal cycler with a heated lid. The presence and absence of specific PCR products were documented using the UV transilluminator. Interpretations of the typing were done with the lot-specific interpretation and specificity tables. The occurrences of specific HLA alleles observed in the control groups were similar to previous report by others [26]–[28].

### Statistical analysis

The analysis was performed to examine the association between allele prevalence and dengue infection. For each HLA allele, the proportion of DENV infected patients and control subjects with the allele were compared, using allele frequency (AF) values. The degree of association between HLA alleles and disease state was expressed as the odds ratio (OR), which is obtained from standard contingency table analysis by Haldane's modification of Woolf's method. Groups with higher OR value are suggestive of increased risk of infection. The *P* value was determined by using the Pearson chi-square analysis of 2×2 tables with values<0.05 as being significant. Fisher's exact test is a statistical significance test used in the analysis of categorical data where sample sizes are small. The test is used to examine the significance of association between two variables and was used when any value in the cell was <5. *P*-values were further subjected to correction according to the Bonferroni's inequality method (corrected *P*, *Pc*) by multiplying the *P*-values with the number of alleles tested for each locus. The *Pc* value effectively raise the standard of proof needed when a wide range of hypotheses are looked at simultaneously. The SPSS software package SPSS, version 14 (SPSS, Inc., Chicago, Ill.), was used for all analysis.

## Results

### Characteristics of study population

This study was carried out at the UMMC, Kuala Lumpur, Malaysia. We selected 92 patients (41 DF and 51 DHF) with confirmed DENV infection and investigated their HLA alleles associations with the dengue disease. The patients' mean age was 30.18 years (range, 13 to 63), and they were selected from the three ethnic groups in Malaysia. The mean duration of illness was 5 days (range, 4 to 9 days). Dengue IgM positive was detected in the serum samples in 85 patients. The HI titre of <1280 indicates patients with primary infection while those with titres >2560 indicated a secondary infection. For the period of hospital admission, the average maximum hematocrit recorded was 43% (range, 29 to 54%) in both DF and DHF patients. The mean nadir of the platelet count was 56.25×10^6^/ml (range, 7×10^6^ to 134×10^6^/ml) in DF and 47.81 (range, 8×10^6^ to 249×10^6^/ml) in DHF patients. In our healthy adult individuals, dengue IgM and HI titres were <10 indicating there were no asymptomatic DENV infection in these individuals.

### Frequencies of HLA class 1 A and B in Malaysian individuals with dengue viral infections and in control subjects

Table 1 shows the frequencies of the HLA class 1 specificities in our dengue case patients. Among the 21 HLA-A alleles studied, 4 alleles were determined to be at frequencies of more than 5% among the Malay and Chinese patients (A*02, A*11, A*24, and A*33). These four predominant HLA-A alleles in Malay and Chinese dengue patients coincide with the ethnically and geographically matched control groups in this study. There was however one allele (A*03) that was shown to be present at higher levels in the healthy control group (Table 2).

**Table 1:** HLA-A, and -B allele frequencies in Malaysian individuals with dengue virus infection (dengue fever and dengue hemorrhagic fever).

Total Patients		Malay	Chinese	Indian	Total Patients		Malay	Chinese	Indian
HLA	AF (%)	AF (%)	AF (%)	AF (%)	HLA	AF (%)	AF (%)	AF (%)	AF (%)
**A01**	**5.43**	4.55	0	**12.07**	**B07**	**5.98**	**6.06**	1.67	**10.34**
**A02**	**20.65**	**19.70**	**26.67**	**15.52**	**B13**	**9.78**	**12.12**	**6.67**	**10.34**
**A03**	3.26	1.52	0	**8.62**	B14	0.54	0	1.67	0
**A11**	**25.54**	**16.67**	**41.67**	**18.97**	**B15**	**14.67**	**22.73**	**13.33**	**6.9**
**A24**	**25.54**	**34.85**	**18.33**	**22.41**	B18	0.54	0	1.67	0
A26	1.63	1.52	0	3.45	B27	1.09	0	3.33	0
**A30**	2.72	3.03	0	**5.17**	**B35**	**7.61**	4.55	**5**	**13.79**
**A31**	2.17	0	1.67	**5.17**	**B37**	3.80	3.03	3.33	**5.17**
A32	0.54	0	1.67	0	**B38**	**5.43**	**12.12**	3.33	0
**A33**	**7.61**	**12.12**	**10**	0	B39	0.54	0	1.67	0
A34	1.63	4.55	0	0	**B40**	**12.5**	**7.58**	**16.67**	**13.79**
**A68**	3.26	1.52	0	**8.62**	B44	1.63	3.03	0	1.72
					**B46**	**5.43**	4.55	**11.67**	0
					B47	0.54	1.52	0	0
					B48	2.72	4.55	3.33	0
					**B51**	**6.52**	4.55	3.33	**12.07**
					**B52**	**5.43**	4.55	1.67	**10.34**
					B53	1.63	1.52	3.33	0
					**B54**	2.17	1.52	**5**	0
					B55	1.09	1.52	1.67	0
					B56	2.17	0	3.33	3.45
					**B57**	3.80	1.52	0	**10.34**
					**B58**	3.26	3.03	**6.67**	0
					B78	1.09	0	1.67	1.72

HLA = human leukocyte antigen; AF = allele frequency (as percentage).

Bold alleles are the predominant alleles present at frequencies more than 5%.

**Table 2:** HLA-A, and -B allele frequencies in control subjects (ethnically and geographically matched, healthy Malaysian individuals).

Total Controls		Malay	Chinese	Indian	Total Controls		Malay	Chinese	Indian
HLA	AF (%)	AF (%)	AF (%)	AF (%)	HLA	AF (%)	AF (%)	AF (%)	AF (%)
**A01**	**6.84**	3.13	3.13	**14.52**	**B07**	**5.26**	4.69	1.56	**9.68**
**A02**	**20.00**	**14.06**	**26.56**	**19.35**	B08	0.53	0	0	1.61
**A03**	**9.47**	**9.38**	**6.25**	**12.90**	**B13**	**6.32**	3.13	**9.38**	**6.45**
**A11**	**24.21**	**25**	**34.38**	**12.90**	B14	0.53	0	1.56	0
**A24**	**21.58**	**34.38**	**9.38**	**20.97**	**B15**	**12.63**	**15.63**	**7.81**	**14.52**
A26	1.05	0	0	3.23	**B18**	**5.26**	**12.5**	1.56	1.61
A29	0.53	0	0	1.61	B27	1.58	1.56	1.56	1.61
A30	0.53	1.56	0	0	**B35**	**12.11**	**15.63**	**7.81**	**12.9**
A31	1.58	0	3.13	1.61	B37	2.63	3.13	0	4.84
A32	0.53	0	0	1.61	**B38**	4.74	**6.25**	**7.81**	0
**A33**	**10.53**	**9.38**	**17.19**	4.84	B39	2.63	3.13	3.13	1.61
A34	1.05	3.13	0	0	**B40**	**12.11**	**6.25**	**17.19**	**12.9**
**A68**	2.11	0	0	**6.45**	B42	0.53	1.56	0	0
					B44	2.63	3.13	3.13	1.61
					**B46**	4.74	3.13	**10.94**	0
					B47	0.53	0	1.56	0
					B48	0.53	1.56	0	0
					**B51**	**5.79**	4.69	4.69	**8.06**
					**B52**	4.21	4.69	0	**8.06**
					B54	1.05	0	3.13	0
					B55	1.58	1.56	1.56	1.61
					B56	1.58	1.56	0	3.23
					**B57**	2.63	0	1.56	**6.45**
					**B58**	**7.89**	**6.25**	**14.06**	3.23

HLA = human leukocyte antigen; AF = allele frequency (as percentage).

Bold alleles are the predominant alleles present at frequencies more than 5%.

In the Indian patients, eight alleles (A*01, A*02, A*03, A*11, A*24, A*30, A*31 and A*68) were noted to be at frequencies of more than 5% (Table 1) of which six were also present at more than 5% (A*01, A*02, A*03, A*11, A*24 and A*68) in the control group. The other two alleles (A*30 and A*31) were present at lower levels (<5%) in the healthy control group (Table 2).

Taking all dengue patients irrespective of race, 5 alleles were determined to be at frequencies of more than 5% [A*11 (25.54%), A*24 (25.54%), A*02 (20.65%), A*33 (7.61%) and A*01 (5.43%)] (Table 1). These five predominant HLA-A alleles in dengue case patients are also present in matched control groups in this study. There was however one allele (HLA-A*03) that was shown to be present at significantly higher levels in the healthy individuals (Table 2).

Of the 36 HLA-B alleles studied, B*07, B*13, B*15, B*38 and B*40 were the most common alleles found at frequencies greater than 5% in Malay dengue patients (Table 1). Three predominant alleles (B*15, B*38 and B*40) were matched with healthy individuals. There were however two alleles (B*07 and B*13) that were shown to be present at lower levels (<5%) in the Malay healthy individuals. Consequently, we also noted three other alleles (B*18, B*35 and B*58) were present more than 5% of allele frequencies in control group in this population (Table 2).

Among the Chinese patients, B*13, B*15, B*35, B*40, B*46, B*54 and B*58 were the most predominant alleles (Table 1). These alleles were also common in the Chinese control group with the exception for HLA-B*54. The allele HLA-B*38 was noted to be present at more than 5% in healthy group but not in this group of patients (Table 2).

For the Indian patients, the B*07, B*13, B*15, B*35, B*37, B*40, B*51, B*52 and B*57 were the most predominant alleles (Table 1). These alleles were also present at frequencies of more than 5% except for HLA-B*37 in the Indian healthy group (Table 2). Overall in all the patients, B*15(14.67%), B*40(12.50%), B*13(9.78%), B*35(7.61%), B*51(6.52%), B*07(5.98%), B*38(5.43%), B*46(5.43%) and B*52(5.43%) were the most common alleles found at higher levels in dengue patients and this coincides with the control groups, with the exception of B*38, B*46 and B*52, which found to be lower (<5%) in controls. It was noted however that HLA-B*18 and B*58 alleles were present at higher levels in healthy individuals. These predominant alleles have also been reported by others in Malaysia [26]–[28].

In this study, we then further analyzed all the alleles without excluding the rare alleles as these alleles may also contribute to the disease associations.

### Positive associations of HLA-A with DF and DHF

Table 3 and 4 show the positive associations of both HLA-A and HLA-B alleles in the different races for both DF and DHF patients. As seen HLA-A*30 allele frequency was increased 2.3 fold, (*P* = 0.518, OR = 0.43, 95% CI = 0.03–7.11, Table 3) in Malay DF patients while HLA-A*26 and HLA-A*68 alleles were increased 2.6 fold in Malay DHF patients (*P* = 0.373, Table 4) as compared to matched controls. For the Chinese group, no associations (>2 fold increase in AF) of HLA-A alleles were observed between the DF patients and controls. However, HLA-A*24 increased 2.0 fold (*P* = 0.206, 0R = 0.45, 95% CI = 0.13–1.52) and HLA-A*32 increased 3.1 fold (*P* = 0.333) in DHF patients as compared to healthy individuals in this population (Table 4). In the Indian patients, increased frequencies of HLA-A*68 (2.4 fold rise, *P* = 0.658, 0R = 0.38, 95% CI = 0.09–1.65) were noted in DF patients as compared to controls (Table 3) while for DHF Indian patients, HLA-A*30 (6.3 fold rise, *P* = 0.113) and A*31 (3.9 fold rise, *P* = 0.266, 0R = 0.25, 95% CI = 0.02–2.82) alleles were observed to be increased in this population compared to healthy controls (Table 4).

**Table 3:** Positive associations of HLA alleles in DF patients in different races.

Alleles	AF%	AF%	Fold	*P*-value	OR	95% CI
	(PT)	(CTRL)				
**Malay (n = 14)**						
A*30	3.57	1.56	2.3	0.518	0.43	0.03–7.11
B*13	7.14	3.13	2.3	0.582	0.42	0.06–3.14
B*37	7.14	3.13	2.3	0.582	0.42	0.06–3.14
B*40	14.3	6.25	2.3	0.240	0.40	0.09–1.73
B*46	7.14	3.13	2.3	0.582	0.42	0.06–3.14
B*48	3.57	1.56	2.3	0.518	0.43	0.03–7.11
**Chinese (n = 14)**						
B*15	21.43	7.81	2.7	**0.084**	0.31	0.09–1.12
B*18	3.57	1.56	2.3	0.518	0.43	0.03–7.11
B*48	3.57	0	3.6	0.304	NA	NA
B*78	3.57	0	3.6	0.304	NA	NA
**Indian (n = 13)**						
A*68	15.38	6.45	2.4	0.658	0.38	0.09–1.65
B*51	23.08	8.06	2.9	**0.076**	0.29	0.08–1.06
B*56	7.69	3.23	2.4	0.578	0.40	0.05–3.00
B*78	3.85	0	3.9	0.295	NA	NA
**Total Patients (n = 41)**						
A*30	2.43	0.53	4.6	0.217	0.21	0.02–2.37
A*68	4.88	2.11	2.3	0.248	0.42	0.10–1.72
B*48	2.44	0.53	4.6	0.217	0.21	0.02–2.37
B*78	2.44	0	2.4	**0.09**	NA	NA

n = number of patients; *P* = *p* value derived from fisher exact test; OR = Odds Ratio; CI = confidence interval; PT = patients; CTRL = controls; AF = allele frequency in percentage; Number in bold indicate nearing significant *p* value; In Pearson chi-square analysis, where a value in a 2×2 table was 0, the OR and 95% CI could not be calculated (NA, not available).

**Table 4:** Positive associations of HLA alleles in DHF patients in different races.

Alleles	AF%	AF%	Fold	*P*-value	*Pc*-value	OR	95% CI
	(PT)	(CTRL)					
**Malay (n = 19)**							
A*26	2.63	0	2.6	0.373		NA	NA
A*68	2.63	0	2.6	0.373		NA	NA
B*13	15.79	3.13	5.1	0.049*	1.176	0.18	0.03–0.90
B*38	13.16	6.25	2.1	0.287		0.44	0.11–1.75
B*48	5.26	1.56	3.4	0.554		0.29	0.03–3.26
B*53	2.63	0	2.6	0.373		NA	NA
B*54	2.63	0	2.6	0.373		NA	NA
**Chinese (n = 16)**							
A*24	18.75	9.38	2.0	0.206		0.45	0.13–1.52
A*32	3.13	0	3.1	0.333		NA	NA
B*07	3.13	1.56	2.0	1		0.49	0.03–8.13
B*14	3.13	1.56	2.0	1		0.49	0.03–8.13
B*27	6.25	1.56	4.0	0.257		0.24	0.02–2.73
B*37	6.25	0	6.3	0.109		NA	NA
B*48	3.13	0	3.1	0.333		NA	NA
B*52	3.13	0	3.1	0.333		NA	NA
B*53	6.25	0	6.3	0.109		NA	NA
B*56	6.25	0	6.3	0.109		NA	NA
**Indian (n = 16)**							
A*30	6.25	0	6.3	0.113		NA	NA
A*31	6.25	1.61	3.9	0.266		0.25	0.02–2.82
**Total Patients (n = 51)**							
A*30	2.94	0.53	5.5	0.124		0.18	0.02–1.70
B*48	2.94	0.53	5.5	0.124		0.18	0.02–1.70
B*53	2.94	0	2.9	0.042*	1.008	NA	NA

n = number of patients; *P* = *p* value derived from fisher exact test; *Pc* = corrected *P* value; OR = Odds Ratio; CI = confidence interval; PT = patients; CTRL = controls; AF = allele frequency in percentage; Number in bold (*) indicate significant *P* value; In Pearson chi-square analysis, where a value in a 2×2 table was 0, the OR and 95% CI could not be calculated (NA, not available).

Taking the total dengue cases in all the three ethnic groups, the HLA-A*68 allele was increased in frequency among DF patients only when compared to control group in our population (2.3 fold rise, *P* = 0.248, 0R = 0.42, 95% CI = 0.10–1.72). The HLA-A*30 allele on the other hand was noted to be present at increased frequencies in both DF (4.6 fold rise, *P* = 0.217, 0R = 0.21, 95% CI = 0.02–2.37) and DHF (5.5 fold rise, *P* = 0.124, 0R = 0.18, 95% CI = 0.02–1.70) patients in the population.

### Positive associations of HLA-B with DF and DHF

As depicted in Table 3 and 4, positive associations were also seen for HLA-B alleles in DENV infected patients. In the Malay patient group a 2.3 fold rise in HLA-B*40 (*P* = 0.24, 0R = 0.4, 95% CI = 0.09–1.73), B*37and B*46 (*P* = 0.582, 0R = 0.419, 95% CI = 0.06–3.14) alleles were detected in DF patients compared to controls. For the DHF group HLA-B*38 allele increased 2.1 fold (*P* = 0.287, 0R = 0.44, 95% CI = 0.11–1.75) and B*53 and B*54 alleles increased 2.6 fold (*P* = 0.373) as compared to healthy controls in this population. Taking both DF and DHF patients among the Malays increased frequencies of HLA-B*13 (2.3 fold rise, *P* = 0.582, 0R = 0.42, 95% CI = 0.06–3.14 in DF; 5.1 fold rise, *P* = 0.049, *Pc* = 1.176; 0R = 0.18, 95% CI = 0.03–0.90 in DHF) and B*48 (2.3 fold rise, *P* = 0.518, 0R = 0.43, 95% CI = 0.03–7.11 in DF; 3.4 fold rise, *P* = 0.554, 0R = 0.29, 95% CI = 0.03–3.26 in DHF) alleles were noted.

In the Chinese DF patients, HLA-B*15 increased 2.7 fold (*P* = 0.084, 0R = 0.31, 95% CI = 0.09–1.12), B*18 increased 2.3 fold (*P* = 0.518, 0R = 0.43, 95% CI = 0.03–7.11) and B*78 increased 3.6 fold (*P* = 0.304) while in the Chinese DHF patients rising frequencies of HLA-B*07, B*14 (2.0 fold rise, *P* = 1, 0R = 0.49, 95% CI = 0.030–8.13), B*27 (4.0 fold rise, *P* = 0.257, 0R = 0.24, 95% CI = 0.02–2.73), B*37, B*53, B*56 (6.3 fold rise, *P* = 0.109) and B*52 (3.1 fold rise, *P* = 0.333) alleles were noted as compared to healthy individuals. In addition, HLA-B*48 (3.6 fold rise, *P* = 0.304 in DF; 3.1 fold rise, *P* = 0.333 in DHF) allele was increased in both patient groups.

Among the Indian DF population, HLA-B*51 increased 2.9 fold (*P* = 0.076, 0R = 0.29, 95% CI = 0.08–1.06), B*56 increased 2.4 fold (*P* = 0.578, 0R = 0.40, 95% CI = 0.05–3.00) and B*78 increased 3.9 fold (*P* = 0.295) as compared to healthy controls. However, there were no associations of HLA-B alleles were noted in between the DHF patients and controls in this population.

Taking the total dengue cases in all the three ethnic groups, we noticed increased frequencies of HLA-B* 78 (2.4 fold rise, *P* = 0.09) allele in DF patients when compared to healthy controls while in the DHF group HLA-B*53 increased 2.9 fold (*P* = 0.042, *Pc* = 1.008) as compared to healthy individuals. We also observed increases in the HLA-B*48 allele in both DF (4.6 fold rise, *P* = 0.217, 0R = 0.21, 95% CI = 0.02–2.37) and DHF (5.5 fold rise, *P* = 0.124, 0R = 0.18, 95% CI = 0.02–1.7) patients.

### Negative HLA-A and B associations with DF and DHF

Table 5 and 6 show the negative associations of HLA-A and B in the various ethnic groups of dengue disease patients. Within the Malay and Chinese DF patient group there were no negative associations of HLA-A alleles were observed (Table 5). However in the Indian DF patients a 2.5 fold decrease was noted with HLA-A*02 allele (*P* = 0.216, 0R = 2.89, 95% CI = 0.60–13.9). For the DHF group however, the HLA-A03 allele frequency was observed to decrease in the three different racial groups (Malay: 9.4 fold decrease, *P* = 0.082; Chinese: 6.3 fold decrease, *P* = 0.298; Indian: 2.1 fold decrease, *P* = 0.486). Taking DHF patients in total this trend was also observed (4.8 fold decrease, *P* = 0.015, 0R = 5.23, 95% CI = 1.19–23.02). Two other alleles (HLA-A*01 and A*31: 3.1 fold decrease, *P* = 0.551) were found to be absent in the Chinese DHF patient group, while in the Indian DHF patient group HLA-A*33 (4.8 fold decrease, *P* = 0.549) and HLA-A*68 alleles were noted to be present at decreased levels (2.1 fold decrease, *P* = 0.658, 0R = 2.14, 95% CI = 0.23–19.97).

**Table 5:** Negative associations of HLA alleles in DF patients in different races.

Alleles	AF%	AF%	Fold	*P*-value	OR	95% CI
	(PT)	(CTRL)				
**Malay (n = 14)**						
B*18	0	12.5	12.5	0.101	NA	NA
B*35	3.57	15.63	4.4	0.163	5	0.61–41.1
B*39	0	3.13	3.1	1	NA	NA
**Chinese (n = 14)**						
B*44	0	3.13	3.1	1	NA	NA
**Indian (n = 13)**						
A*02	7.69	19.35	2.5	0.216	2.89	0.60–13.9
B*52	3.85	8.06	2.1	0.665	2.19	0.24–19.75
**Total Patients (n = 41)**						
B*35	4.88	12.11	2.5	**0.078**	2.69	0.90–8.03

n = number of patients; *P* = *p* value derived from fisher exact test; OR = Odds Ratio; CI = confidence interval; PT = patients; CTRL = controls; AF = allele frequency in percentage; Number in bold indicate nearing significant *P* value; In Pearson chi-square analysis, where a value in a 2×2 table was 0, the OR and 95% CI could not be calculated (NA, not available).

**Table 6:** Negative associations of HLA alleles in DHF patients in different races.

Alleles	AF%	AF%	Fold	*P*-value	*Pc*-value	OR	95% CI
	(PT)	(CTRL)					
**Malay (n = 19)**							
A*03	0	9.38	9.4	**0.082**		NA	NA
B*18	0	12.5	12.5	0.024*	0.576	NA	NA
B*35	5.26	15.63	2.9	0.202		3.33	0.69–16.11
B*37	0	3.13	3.1	0.528		NA	NA
B*39	0	3.13	3.1	0.528		NA	NA
B*40	2.63	6.25	2.4	0.648		2.47	0.27–22.92
B*58	2.63	6.25	2.4	0.648		2.47	0.27–22.92
**Chinese (n = 16)**							
A*03	0	6.25	6.3	0.298		NA	NA
A*01	0	3.13	3.1	0.551		NA	NA
A*31	0	3.13	3.1	0.551		NA	NA
B*13	0	9.38	9.4	0.174		NA	NA
B*39	0	3.13	3.1	0.551		NA	NA
B*44	0	3.13	3.1	0.551		NA	NA
B*58	6.25	14.06	2.3	0.327		2.46	0.50–12.10
**Indian (n = 16)**							
A*03	6.25	12.9	2.1	0.486		2.22	0.44–11.15
A*33	0	4.84	4.8	0.549		NA	NA
A*68	3.13	6.45	2.1	0.658		2.14	0.23–19.97
B*15	6.25	14.52	2.3	0.322		2.55	0.52–12.57
B*51	3.13	8.06	2.6	0.66		2.72	0.30–24.33
B*56	0	3.23	3.2	0.55		NA	NA
B*58	0	3.23	3.2	0.546		NA	NA
**Total Patients (n = 51)**							
A*03	1.96	9.47	4.8	0.015*	0.18	5.23	1.19–23.02
B*18	0	5.26	5.3	0.017*	0.408	NA	NA
B*58	3.26	7.89	2.7	0.126		2.83	0.80–10.01

n = number of patients; *P* = *p* value derived from fisher exact test; *Pc* = corrected *P* value; OR = Odds Ratio; CI = confidence interval; PT = patients; CTRL = controls; AF = allele frequency in percentage; Number in bold indicate (*) significant *P* value; Number in bold indicate nearing significant *P* value In Pearson chi-square analysis, where a value in a 2×2 table was 0, the OR and 95% CI could not be calculated (NA, not available).

For the HLA-B allele group (Table 5 and 6), distinctively in the Malay patients, decreased frequencies of alleles HLA-B*18 (12.5 fold decrease, *P* = 0.101 in DF; 12.5 fold decrease, *P* = 0.024, *Pc* = 0.576 in DHF), B*35 (4.4 fold decrease, *P* = 0.163, OR = 5, 95% CI = 0.61–41.1 in DF; 2.9 fold decrease, *P* = 0.202, 0R = 3.33, 95% CI = 0.69–16.11 in DHF) and B*39 (3.1 fold decrease, *P* = 1 in DF; 3.1 fold decrease, *P* = 0.528 in DHF) were observed both in DF and DHF patients. In addition, HLA-B*37 (3.1 fold decrease, *P* = 0.528 in DHF), B*40 and B*58 (2.4 fold decrease, *P* = 0.648, 0R = 2.47, 95% CI = 0.27–22.92 in DHF) alleles were decreased in frequencies in DHF patients only in this population group. In the Chinese population, absence of HLA-B*13 (9.4 fold decrease, *P* = 0.174), B*39(3.1 fold decrease, *P* = 0.551) and decreased frequencies of HLA-B*58 (2.3 fold decrease, *P* = 0.327, 0R = 2.46, 95% CI = 0.50–12.10) alleles were detected in DHF patients compared in study controls. Further, HLA-B*44 (3.1 fold decrease, *P* = 1 in DF, 3.1 fold decrease, *P* = 0.551 in DHF) allele was observed to be decreased in both dengue case groups. Among the Indian DF patients, HLA-B*52 (2.1 fold decrease, *P* = 0.665, 0R = 2.19, 95% CI = 0.24–19.75) allele was noted to be decreased in frequency. Lower levels of the HLA-B*15 (2.3 fold decrease, *P* = 0.322, 0R = 2.55, 95% CI = 0.52–12.57), B*51 (2.6 fold decrease, *P* = 0.66, 0R = 2.72, 95% CI = 0.30–24.33), B*56 (3.2 fold decrease, *P* = 0.55) and B*58 (3.2 fold decrease, *P* = 0.546) alleles were also detected in DHF patients in this group as compared to healthy donors.

Taking all the ethnic groups as a whole, lower levels of HLA-B*35 (2.5 fold decrease, *P* = 0.078, 0R = 2.69, 95% CI = 0.90–8.03) allele was noted in DF patients while two other alleles (HLA-B*18 : 5.3 fold decrease, *P* = 0.017, *Pc* = 0.408 and B*58 : 2.68 fold decrease, *P* = 0.126, 0R = 2.83, 95% CI = 0.80–10.01) were noted to decrease in DHF patients as compared to controls.

### HLA-A24: An association with dengue viral infection

HLA-A*24 is an allele very frequently expressed especially in the DHF and DSS patients [29]. In this study, higher frequencies of HLA-A*24 was found to be present largely in healthy and DENV infected individuals in the Malay and Indian population groups. As stated earlier, in the Chinese population, the presence of this allele was noted to be higher in the DHF patients as compared to healthy controls. We then subtyped the HLA-A*24 alleles in order to identify underlying associations of this allele to dengue disease in this population. There is evidence that subtypes may be negatively or positively associated with dengue disease severity [30]. In this present study, 4 different A*24 alleles, A*2401, A*2402, A*2407 and A*2410 were identified (Table 7). Interestingly we found, the presence of HLA-A*2401 and HLA-A*2410 allele frequencies were lower by 7.3 and 2.5 fold respectively in all dengue case patients compared to healthy individuals. However, the frequency level of HLA-A*2402 allele was noted to be increased but only at 1.17 fold in the patients compared to controls.

**Table 7:** Allele Frequencies of HLA-A*24 subtypes in DENV infected patients and healthy individuals.

	Healthy Donor		Patients (DF+DHF)	
HLA-A*24	n = 41	AF (%)	n = 20	AF (%)
2401	3	7.3	0	0
2402	21	51.2	12	60
2407	16	39	8	40
2410	1	2.5	0	0

n = number of patients; HLA = human leukocyte antigen; AF = allele frequency (as percentage); DF = dengue fever; DHF = dengue hemorrhagic fever.

## Discussion

Malaysia has a warm humid climate and a dense population in urban areas. Both rural and urban Malaysian populations are seasonally at risk of DENV infection [14]. The different ethnic groups make this country rich in genetic diversity. HLA classification with its extensive polymorphism is an excellent marker for population genetic analysis and disease association studies [30]. This is the first study on HLA determinants of susceptibility and/or protection to DENV infection in a Malaysian population.

The results obtained, reveal that, HLA-A*30 occurred more frequently among the overall Malaysian DHF population group. However, when we cluster our population into the different racial groups, positive associations were noted with this allele and HLA-A*31 only in the Indian DHF patients. Among the Malay DHF patients, positive association were seen with HLA-A*26 and A*68 alleles while in the Chinese DHF patients, positive associations were noted with HLA-A*24 and A*32 alleles. Although, we observed positive associations, they were not statistically significant. However with an AF of >2 fold rise in DHF patients as compared to the healthy controls, this may suggest a probable risk association to DENV infections. It has been shown that HLA-A*24 was significantly associated with dengue disease severity in Vietnamese population [31]. Others, have also recently demonstrated that the subtype HLA-A*2402 was found significantly higher in the DHF and DSS patients [29]. This further supports our findings, where higher expression of HLA-A*2402 was seen in our patients than in controls which implies a probable risk association with severe disease in the Malaysian population too. However, the obtained *P* value here was not significant and this could probably be due to the lower number of samples. Risk associations of HLA-A*30 and A*68 alleles have been reported for Human Immunodeficiency Virus (HIV-Type 1) in Southern Africans [32]. In Japanese HIV-1 disease patients, HLA-A*26 allele has been significantly associated with a slow progression to AIDS [33] while HLA-A*31 allele has been linked with epithelial cancer patients in Japan [34].

In the present study protective association of allele HLA-A*03 was observed for the first time in DHF patients in the pooled ethnic population. When stratified by race, a stronger negative association trend was identified especially in the Malay patient group as compared to Chinese and Indian DHF patients. Currently no association of this allele has been demonstrated by others in dengue diseases, yet this allele has been shown to play an essential role in elimination of Hepatitis B Virus (HBV) and Hepatitis C Virus (HCV) respectively in the Caucasian and Irish populations [35], [36]. Also it has been reported that those carrying the HLA-A*03 allele are negatively associated with Posttransplant lymphoproliferative disease (PTLD) [37]. In the Chinese population, the HLA-A*01 was found to be negatively associated with DHF. Our finding is in contrast to that of the Cuban study where this allele was shown to be positively associated with susceptibility to DHF [20]. Other studies have suggested the increased frequency of this allele in patients with primary dengue infection [19] and its probable association in the development of severe clinical disease [20]. Interestingly, within our population study group, a probable reverse association (protective to susceptible) of HLA-A*31 allele is seen between Chinese and Indian DHF patients and HLA-A*68 allele between Indian and Malay DHF patients. The role of HLA-A*68 in facilitating the rapid progression of pleural disease and to allow the replication of mycobacteria has been documented in HIV-positive patients [38].

Analysis of the HLA-B alleles in different ethnic groups suggests a possible significant association of allele B*13 with disease susceptibility, in the Malay DHF population. However, this result is probably in contrast to the result obtained with Chinese DHF population in this present study and previous study in Thai populations, where allele B*13 was associated with protection to dengue viral infections [39]. The varying frequencies of these alleles in different populations may account, to some extent, for increased resistance and/or susceptibility to diseases in different populations.

The higher frequency of alleles B*38, B*48, B*53, B*54 and complete absence or lower frequencies of alleles B*37, B*39, B*35, B*40, B*58 in Malay DHF patients, as compared with controls, were not definitively associated with susceptibility or protection to dengue disease, as obtained *P* and *Pc*-values were not significant. However, interestingly we noticed a significant decrease of allele B*18 in the Malay DHF patients compared to control group. This may suggest the probable protective role of this allele towards DHF patients in this population. However, others have documented the risk association of HLA-B*18 in Malay patients with nasopharyngeal carcinoma [40]. Furthermore, a probable protective association of HLA-B*18 allele have been reported in breast feeding infants against HIV-1 acquisition [41]. Besides, two different studies have shown, the association of HLA-B*54 allele with a greater risk of developing myelopathy caused by type 1 human T-cell lymphotropic virus (HTLV-1) [42], [43].

In the Chinese DHF patients, the presence of alleles HLA-B*07, B*14, B*27, B*37, B*48, B*52, B*53, B*56 at the higher frequencies and HLA-B*39, B*44, B*58 at the lower frequencies compared to healthy donors remains uncertain as *P* values were also not significant. However, others have reported the significant role of these alleles in their study. For an example, the allele B*07, was found to be low in mainland Southeast Asians [44] and a study done on Thais showed that T cell responses to an HLA-B*07-restricted epitope on the dengue NS3 antigens correlate with disease severity [45]. In addition, the HLA-B*14 and B*52 alleles were reported to be protective against dengue disease respectively in Cuban and Thai populations [20], [39], which are probably in contrast to our present finding. The HLA-B*44 was on the other hand reported to be protective against dengue disease in the Thais [39]. Recently, HLA-B*27 was documented to have a protective association against infectious agents such as HIV and HCV [46]–[48].

Lower allele frequencies of alleles B*15, B*51, B*56, B*58 in the Indian DHF patients as compared with ethnically matched controls, remain undefined as well, as obtained *P*-values were also not significant. A study in ethnic Thais confirmed the association of allele B*51, with clinical outcome, in previously dengue exposed and immunologically primed individuals [39]. The involvement of HLA-B*51-restricted CTL responses to a variety of viruses have been reported, including for Hantaan virus which also causes a hemorrhagic fever [49]. However, the variation in our results on allele B*51 which may suggest probable protection role in dengue viral infection, in comparison to the other populations could be due to their distinctly diverse genetic background.

In this study the *P* value just reached significant levels for HLA-A*03, B*13 and B*18 before Bonferoni correction. Nevertheless, it is essential to verify that the loss of significance might probably be as a consequence, exclusively of the rigor of statistical analysis, as the *P*-values without correction were obtained to be significant. An independent study with a larger number of patient samples may probably resolve this issue.

Previous cohort studies by others have reported that, DF and DHF patients show significant diversity in allele frequency to each other or to control, signifying that DF and DHF involve genetically distinct immune response. This support our findings too, where in the DF group specifically, the alleles HLA-A*68 and HLA-B*78 were increased in frequencies (nearing significant; *P* = 0.09). In the Malay population, the presence of HLA-A*30, B*37, B*40 and B*46 were noticed more than two fold rise in DF patients compared to healthy individuals. In Chinese DF patients, increased in HLA-B*15 (nearing significant; *P* = 0.084), B*18 and B*78 and in the Indian DF patients increased in HLA-A*68, B*51 (nearing significant; P = 0.076), B*56 and B*78 were seen compared to controls respectively. In the negative association of HLA alleles in DF patients, the allele frequency of HLA-B*35(nearing significant; P = 0.078) was seen to be decreased in overall population and specifically HLA-A*02 and B*52 in Indian DF patients. A recent study have documented the negative association of HLA-B*35 in Mexican Mestizo patients with DF. Though the presence of these alleles does not differ significantly in DF patients compared to control, yet, interestingly as mentioned earlier we do also notice the expression of different allele with the same trend of allele frequencies in our DF and DHF group [50].

The HLA-mediated restriction of the T cell response to dengue viruses has been repeatedly documented to promote different infection outcomes. The correlation of HLA-A*03 with CD8 T cell responses has been determined in our previous study [51]. Five peptides presented by HLA-A*03 was found to be negatively associated with dengue disease. These 5 peptides (1 peptide from NS2B protein, 2 peptides from NS3 protein and 1 peptide from NS4B and NS5 proteins) belong to the non-structural region of the DENV and were associated with secondary DENV-1 infections. One of the peptides RVIDPRRCMK_422–431_ from NS3 region of the DENV-2 was recognized by patients from different ethnics (Malay, Chinese and Indian) in our population possessing different type of HLA alleles. This could probably be the immunodominant peptide as it possesses the highest T cell responses in comparison with the other four recognized peptides.

In conclusion, the genetic variations among our study population have expanded our knowledge of the alleles that contribute to risk and/or protection of dengue viral infection in the three ethnic groups in Malaysia. Further studies are definitely required to assess these potential associations in a larger population group and to understand whether these associations result from individual genotype or the haplotypes. Notably, the genetic documentations, in support with previous reports by others, with specific associations of HLA molecules with dengue disease will be an essential tool in identifying the dengue specific epitopes presented by susceptible and/or protective alleles. This fundamental data also contributes in further understanding dengue immunopathogenesis and relevance for DENV vaccine design in our population.
