# Correction: Susceptible and Protective HLA Class 1 Alleles against Dengue Fever and Dengue Hemorrhagic Fever Patients in a Malaysian Population

**DOI:** 10.1371/annotation/972cc8f0-5c9e-4d74-b98b-217a2f80a4bd

**Published:** 2011-01-27

**Authors:** Ramapraba Appanna, Sasheela Ponnampalavanar, Lucy Lum Chai See, Shamala Devi Sekaran

An incorrect number was entered in the Funding section. "University Malaya Vote PJP [FS245/2008B]" should be: "University Malaya HIR [J-00000-73560.]"

